# Detection of interleukin-22 secreting T-cells in piperacillin hypersensitive patients with cystic fibrosis

**DOI:** 10.1186/2045-7022-4-S3-P108

**Published:** 2014-07-18

**Authors:** Andrew Sullivan, Eryi Wang, John Farrell, Paul Whitaker, Daniel Peckham, B Kevin Park, Dean Naisbitt

**Affiliations:** 1 University Of Liverpool, Centre for Drug Safety Science, UK; 2 St James' Hospital, Regional Adult Cystic Fibrosis Unit, UK

## 

Delayed-type cutaneous drug hypersensitivity is a T-cell mediated disease; the spectrum of conditions has been classified according to the phenotype and function of drug antigen-specific T-cells sub-sets. Importantly, this classification does not take into account tissue-specific cytokines such as interleukin-17 and interleukin-22. Both of these cytokines have been shown to play a role in numerous physiological and pathological processes. For example, elevated levels have been detected in patients with psoriasis and allergic contact dermatitis. To investigate this, we have focused on piperacillin, a β-lactam antibiotic used for the treatment of pulmonary exacerbations in patients with cystic fibrosis. Unfortunately hypersensitivity reactions (mean onset = 9.1 days; symptoms include maculopapular rash, fever and/or flu-like symptoms) to this drug develop in 26-50% of treated patients. Piperacillin-specific T-cell clones were generated from 3 lymphocyte transformation test positive hypersensitive patients and 4 healthy donors, following priming of naïve T-cells against piperacillin-exposed dendritic cells, to characterize T-cell phenotype (CD and chemokine receptor expression) and function (proliferation and secretion of cytokines/cytolytic molecules [IFNγ, 13, 17, 22 by ELIspot]). Seventy six CD4+ and CD8+ clones were isolated from the hypersensitive patients and shown to proliferate in the presence of piperacillin. Drug stimulation was associated with the secretion of IFN-γ and IL13 from all clones. IL-22 secretion was detected from 64% and 75% of the CD4+ and CD8+ clones, respectively. In contrast, IL17 was not detected. Naïve T-cells co-cultured with piperacillin and autologous dendritic cells showed concentration-dependent proliferation and cytokine (IFNγ, IL13 and IL22) secretion. CD4+ and CD8+ clones generated from the primed T-cells also secreted IFNγ, IL13 and IL22 (CD4+ 50%, CD8+ 67%) following piperacillin stimulation. CCR1, CCR4 and CCR10 were expressed on all clones. Other chemokine receptors expressed on a limited number of clones included CXCR3, CXCR6, CCR2 and CCR9. In conclusion, these data show the involvement of IL22 secreting T-cells in the pathogenesis of piperacillin hypersensitivity reactions in patients with cystic fibrosis.

